# Iowa Gambling Task (IGT): twenty years after – gambling disorder and IGT

**DOI:** 10.3389/fpsyg.2013.00665

**Published:** 2013-09-30

**Authors:** Damien Brevers, Antoine Bechara, Axel Cleeremans, Xavier Noël

**Affiliations:** ^1^Department of Medicine, Psychological Medicine Laboratory, Faculty of Medicine, Université Libre de BruxellesBrussels, Belgium; ^2^Department of Psychology, Brain and Creativity Institute, University of Southern CaliforniaLos Angeles, CA, USA; ^3^Department of Psychology, Consciousness, Cognition & Computation Group, Center for Research in Cognition & Neuroscience, Université Libre de BruxellesBrussels, Belgium

**Keywords:** gambling disorder, Iowa Gambling Task, decision-making, dual-process model, willpower

## Abstract

The Iowa Gambling Task (IGT) involves probabilistic learning via monetary rewards and punishments, where advantageous task performance requires subjects to forego potential large immediate rewards for small longer-term rewards to avoid larger losses. Pathological gamblers (PG) perform worse on the IGT compared to controls, relating to their persistent preference toward high, immediate, and uncertain rewards despite experiencing larger losses. In this contribution, we review studies that investigated processes associated with poor IGT performance in PG. Findings from these studies seem to fit with recent neurocognitive models of addiction, which argue that the diminished ability of addicted individuals to ponder short-term against long-term consequences of a choice may be the product of an hyperactive automatic attentional and memory system for signaling the presence of addiction-related cues (e.g., high uncertain rewards associated with disadvantageous decks selection during the IGT) and for attributing to such cues pleasure and excitement. This incentive-salience associated with gambling-related choice in PG may be so high that it could literally “hijack” resources [“hot” executive functions (EFs)] involved in emotional self-regulation and necessary to allow the enactment of further elaborate decontextualized problem-solving abilities (“cool” EFs). A framework for future research is also proposed, which highlights the need for studies examining how these processes contribute specifically to the aberrant choice profile displayed by PG on the IGT.

## Introduction

Gambling, defined as an activity in which something of value is risked on the outcome of an event when the probability of winning or losing is less than certain (Korn and Shaffer, 1999), is a very popular recreational activity. Between 50 and 80% of the general population gamble at least one time per year (e.g., Abbott and Volberg, 1995; Welte et al., 2002). However, for some individuals (about 15% of frequent gamblers and about 1.6% of the general population; Wardle et al., 2007; INSERM, 2008), gambling can spiral out of control and become a financial burden on the individual and his/her family.

Gambling disorder is defined as persistent and recurrent maladaptive gambling behavior characterized by an inability to control gambling that disrupts personal, family or vocational pursuits (APA, 2013). More specifically, similar to substance (e.g., alcohol, cocaine) addictions, pathological gamblers (PG) exhibit a loss of willpower to resist gambling: they persist in gambling despite the occurrence of negative consequences (e.g., loss of a significant relationship, job or career opportunity) (APA, 2013).

Over the last decade, research has focused on the neurocognitive determinants of gambling disorder and found a number of similarities between drug addiction and gambling addiction (for a review, see Leeman and Potenza, 2012), suggesting that gambling addiction shares common mechanisms with substance addiction. These findings are in line with the new classification of gambling disorder in the DSM-V (APA, 2013), which views gambling disorder as a “behavioral addiction” that, unlike substance abuse, does not involve intake of an exogenous substance. Hence, given the absence of the confounding effect of chemical substances that can alter the brain in many non-specific ways, the study of gambling disorder offers one critical approach to understand and extract components specifically involved in the development of addiction.

With respect to the study of impaired decision-making in addiction, the Iowa Gambling Task (IGT; Bechara et al., 1994) has been regarded as the most widely used and ecologically valid measure of decision making in this clinical population. One of the reasons for this ecological validity is that performing advantageously on this task requires, as in real-life, dealing with uncertainty in a context of punishment and reward, with some choices being advantageous in the short-term (high reward), but disadvantageous in the long run (higher punishment); other choices are less attractive in the short-term (low reward), but advantageous in the long run (lower punishment). Hence, the key feature of this task is that participants have to forgo short-term benefits for long-term benefits, a process that is presumably severely hampered in drug and gambling addicts (APA, 2013). Accordingly, performance on the IGT has been shown to be a sensitive measure of impaired decision-making in a diversity of neurological and psychiatric conditions (Bechara, 2007). For instance, patients with frontal lesions (Bechara et al., 1994, 2000; Manes et al., 2002) and substance dependent (SD) individuals (Petry et al., 1998; Grant et al., 2000; Bechara et al., 2001; Whitlow et al., 2004) have demonstrated a preference for short-term gains despite larger net losses while performing the IGT. With regard to PG, it also appears that they display a stubborn preference for disadvantageous deck selection during the IGT (see Table 1).

**Table 1:** **Studies using the IGT in gambling disorder**.

**Study**	**Participants**	**SOGS score *(SD)***	**Cognitive tasks**	**Main results**
Brevers et al., 2012a	PrG ranging from low	DSM diagnose	IGT	IGT, CPT, Cups task, CFT, OSPAN: PrG < HC
	PrG to severe PG	7.07 (3.74)	Card Playing Task (CPT)	
	PG = 65, 50 male		Cups task	Problem gambling severity correlates with performance on the IGT and the CPT
	HC = 35, 29 male		Coin Flipping Task	
			Operation span working memory task (OSPAN)	In HC: correlation between later stages of IGT and OSPAN
				In PG: no correlation between later stages of IGT and OSPAN
Brevers et al., 2013b	PG = 30, 29 male	DSM diagnose	IGT with post-decision wagering	IGT: PG < HC
	HC = 35, 27 male			PG whereas HC
				HC maximized their wagers on advantageous decks and minimized their wagers on disadvantageous decks PG maximized their wagers independently of selecting advantageous decks
Cavedini et al., 2002	PG = 20, 19 male	DSM diagnose	IGT	IGT: PG < HC
	HC = 40, 28 male	15.8 (3.6)	Weigl's Sorting Test (WST)	WST: PG = HC
			Wisconsin Card Sorting Test (WCST)	WCST: PG = HC
De Wilde et al., 2013	PG = 21, 20 male	DSM diagnose	IGT	IGT, DDT, Stroop: PG = HC
	HC = 31, 27 male	11.14 (4.12)	Delay Discounting Task (DDT) Stroop with gambling words	Stroop: PG < HC
Forbush et al., 2008	PG = 25, 14 male	DSM diagnosis	IGT	Stroop, WAIS, WCST, COWAT and BDAEANT: PG < HC
	HC = 34, 9 male		WAIS letter and numbers and picture	Stroop IGT: PG < HC
			Controlled Oral Word Association Test (COWAT)	Trail Making Task A and B: PG = HC
			WCST-64	
			Boston diagnostic aphasia exam animal naming test (BDAEANT) Trail Making Task A and B	
Goudriaan et al., 2005	PG = 48, 41 male	DSM diagnose	IGT	IGT: PG < HC; PG = AD
	AD = 46, 36 male	13.9 (6.3)	Computerized card playing task	IGT perseveration: PG < HC
	TS = 47, 32 male		GO/NO-GO task with reward and loss version	Commission errors GO/NO-GO: PG > HC
Goudriaan et al., 2006	PG = 46, 39 male	DSM diagnose	IGT with skin conductance	IGT: PG < HC
	HC = 47, 36 male	14.4 (6.1)	response (SCR) and heart rate	HR decrease before choosing bad deck in HC < PG
			(HR) reactivity	SCR reaction to disadvantageous decks HC > PG
				HR decreases with loss and increases in wins in HC HR decreases for both wins and losses in PG
Kertzman et al., 2011	PG = 51, 35 male	DSM diagnose	IGT	IGT: PG < HC
	HC = 57, 36 male	14.4 (6.1)	Stroop task	Stroop task, Go/NoGo: PG < HC
			Go/NoGo task	No association between Stroop + Go/NoGO and IGT performance
Lakey et al., 2007	HC = 57, 48 male	DIGS	IGT	Overconfidence and bed acceptance on the GGT and disadvantageous choices on Problem gambling severity correlates with performance on the IGT
	PrG = 85, 63 male	0–2	GGT (overconfidence measures)	
	PG = 79, 55 male	3–4		
		>5		
Ledgerwood et al., 2012	PG = 45, 21 male	NODS lifetime	IGT	IGT: PG < HC
	HC = 45, 23 male	8.0 (1.7)	Tower of London	Tower of London: PG < HC
		NODS past year	GoStop response inhibition task	GoStop, Stroop; COWAT, WCST: PG = HC
		7.5 (1.8)	Stroop test	
			COWAT	
			WCST	
Linnet et al., 2006	PG = 61, 54 male	8.93 (1.86)	IGT (*Mouse Game* version)	IGT: PG < HC
	HC = 39, 11 male			Switching behavior after negative feedback: PG < HC
Linnet et al., 2010	PG = 16, all male	DSM diagnose	IGT (ABCD, KLMN and QRST versions) with PET using [^11^C]raclopride to measure dopamine release in the ventral striatum	PG who lost money (net IGT outcome) significantly increased dopamine release in the left ventral striatum compared with HC
	HC = 15, all male	13.12 (2.06)		PG and HC who won money did not differ in dopamine release
Linnet et al., 2011a	PG = 16, all male	DSM diagnose	IGT (ABCD, KLMN and QRST versions) with PET using [^11^C]raclopride to measure dopamine release in the ventral striatum	IGT: PG = HC
	HC = 14, all male	13.19 (2.11)		Dopamine release was associated with higher IGT performance in HC and significantly lower IGT performance PG
Linnet et al., 2011b	PG = 18, all male	DSM diagnose	IGT (ABCD, KLMN and QRST versions) with PET using [^11^C]raclopride to measure dopamine release in the ventral striatum	PG with dopamine release in the ventral striatum had significantly higher excitement levels than HC despite lower IGT performance
	HC = 16, all male			No differences in excitement levels and IGT performance were found between PG and HC without dopamine release PG showed a significant correlation between dopamine release and excitement level, while no such interaction was found in HC
Linnet et al., 2012	PG = 18, all male	DSM diagnose	IGT with PET using [^11^C]raclopride to measure dopamine release in the ventral striatum	High dopamine release in PG in which the probability of selecting advantageous decks is maximally uncertain (ratio advantageous decisions/total decisions = 0.05)
	HC = 16, all male			
Oberg et al., 2011	PG = 15, all male	NODS	IGT modified version with EEG	IGT: PG < HC
	HC = 13, all male	2.8		HC < PG MedioFrontal Negativity, 185 ms post-disadvantageous deck outcome
		CPGI		PG < HC P300 Theta Amplitude, 300 ms post-disadvantageous deck outcome
		5.4		
Peterson et al., 2010	PG = 11, all male	DSM diagnose	IGT (ABCD, KLMN and QRST versions) with SCR reactivity and PET using [^11^C]raclopride to measure dopamine release	Active IGT gambling minus passive IGT gambling: HC < PG in SCR
	HC = 11, all male			In both PG and HC, highly sensation-seeking subjects had significant increase of receptor availability in striatum, compared to normally sensation-seeking subjects
Petry, 2001	SD = 63, all male	DSM diagnose	IGT	PG + SD < SD < HC
	PG + SD = 27, all male	9.3 (2.8)		
	HC = 21, all male			
Power et al., 2012	PG = 13, all male	DSM diagnose	IGT with fMRI	IGT: PG < HC
	HC = 13, all male	13.00 (4.00)		Bad deck minus bad decks: HC < PG in the orbitofrontal cortex, caudate nucleus and the amygdala
Roca et al., 2008	PG = 11	DSM diagnose	IGT	IGT: HC > PG
	HC = 11		GO/NO-GO	GO/NO-GO: HC < PG
	Unknown ratio		Addenbrooke's cognitive examination; short screen for general cognitive functions	General cognitive functions; word fluency and memory: HC > PG
	male/female			In PG: no association between IGT and other cognitive task
Tanabe et al., 2007	SD = 14, 10 male	10.7 (4.4)	IGT modified version with fMRI	IGT: SD = SD + PG = HC
	SD + PG = 14, 12 male	0.2 (0.4)		Decision making minus control condition: OFC, ventral medial dorsal, ventrolateral/anterior insula, ACC, ventral striatum, parietal en occipital lobes in all groups
				SD = SD + PG < HC in ventral medial prefrontal cortex activity SD < SD + PG = HC in right anterior prefrontal cortex activity

These studies were selected in the basis of a comprehensive literature search conducted in PUBMED and PsychINFO with key search terms, including: Iowa gambling task, IGT, decision making, uncertain^*^, ambig^*^ in combination with the key word gambl^*^. Cross-references were searched in the selected articles. A total of 1387 hits were retrieved in PUBMED and PsychINFO using the search terms. Selection criteria for studies were inclusion of the original or adapted version of the IGT, presence of a gamblers group (ranging from frequent to severe pathological gamblers). After this selection, 28 papers remained, 7 articles were excluded because no control group was included in the study (n = 1) or it concerned review articles (n = 6). SOGS, South Oaks Gambling Screen; HC, healthy controls; PG, pathological gamblers; PrG, problem gambler; SD, substance dependent.

But what are the processes underlying this inability to optimally ponder immediate vs. long terms consequences of a choice (Bechara, 2005)? On the basis of the dual-process model of self-regulation (e.g., Bechara, 2005; Everitt and Robbins, 2005; Redish et al., 2008), the ability to decide advantageously according to short-term and long-term outcomes involves the optimal activation of two neural systems: (i) an “*impulsive*,” amygdala-striatum dependent, neural system that promotes automatic, habitual, and salient behaviors; and (ii) a prefrontal “*reflective*” neural system that forecasts the future consequences of a behavior and allows inhibitory control of automatic responses. The “*impulsive*” system is critical for processing the incentive motivational effects of a variety of natural (e.g., food) and non-natural rewards (e.g., money), which are mainly processed through an amygdala-striatal neural system (Robbins et al., 1989; Wise and Rompre, 1989; Robinson and Berridge, 1993; Di Chiara, 1999). Importantly, this is also the neural system that has been argued to be responsible for the transfer of reward seeking from controlled to automatic and habitual behaviors (Everitt et al., 1999; Everitt and Robbins, 2005). The “*reflective*” system is necessary to control basic impulses and allow the more flexible pursuit of long-term goals. This system includes executive functions (EFs), which could be understood as a variety of cognitive abilities that allow the conscious control of thought, emotion and action. The action of the reflective system depends on the integrity of two sets of neural systems: a “cool” and a “hot” EFs system (Zelazo and Müller, 2002). These “cool” and “hot” EFs are achieved through relatively slow, controlled processes and allow to hold on to a mental representation for contemplation and self-reflection (Smith and DeCoster, 2000). “Cool” EFs are mediated by lateral inferior and dorsolateral frontostriatal and frontoparietal networks and refer to abstract decontextualized reasoning (Kerr and Zelazo, 2004). More specifically, “cool” executive processes include problem-solving abilities that require the capacity to represent a dilemma, maintain, and organize information in working memory, strategically plan and execute a response, evaluate the efficacy of the solution, and make necessary changes based on the outcome (e.g., shifting back and forth between multiple tasks and the ability to deliberately suppress prepotent responses that are no longer relevant) (Zelazo and Müller, 2002). Hence, “cool” EFs is associated with rational and cognitive determinations of risks and benefits associated with options, and requires the knowledge of the risk/benefit ratio, the ability to retrieve them from memory, and the ability hold them in mind while comparing and contrasting them through working memory processes (Seguin et al., 2007). In contrast, “hot” EFs refer to one's ability to monitor the self and the situation for what are considered to be acceptable social behaviors, regulate emotional responses, and inhibit impulsive reactions. These EFs are mediated by ventromedial (VMPC) and orbito (OFC) prefrontal cortex structures that are closely connected to the limbic system, which confers to hot EFs a critical role in regulating affective and motivational processes (Zelazo and Müller, 2002). Hence, by overcoming impulsive triggers, “hot” executive processing results in the ability to advantageously weigh short-term gains against long-term losses, that is, to optimally anticipate the potential outcomes of a given decision (Damasio, 1996). Importantly, several theoretical accounts advance that before elaborate decontextualized problem-solving abilities and other related cognitive skills (i.e., “cool” EFs) can begin to be enacted, the ability to control emotional reactions and inhibit basic behavioral impulses may be required first (Barkley, 1997; Sonuga-Barke et al., 2002; Giancola et al., 2012). More specifically, the ability to control emotional reactions and inhibit basic behavioral impulses by “hot” EFs would allow rational and cognitive determinations of risks and benefits associated with options (Giancola et al., 2012). For instance, when exposed to high-uncertain rewards, individuals with intact “hot” EF capacities will be capable to control their emotional responses and inhibiting their impulses directed at the reward, which will then make it significantly more likely that they will engage in the more cool abstract reasoning/problem-solving aspects of EF. In turn, the enactment of those “cool” EFs would reinforce the efficiency of reward anticipation processes (e.g., to weigh short-term gains against long-term losses on both emotional and rational bases). Thus, adequate decision-making reflects an integration of cognitive (i.e., “cool” EFs) and affective (i.e., “hot” EFs) systems, and the ability to more optimally weigh short term gains against long term losses or probable outcomes of an action. One important consequence of this assumption is that, if learning is suddenly interrupted (e.g., absence of deck selection outcomes during a IGT “blind” phase, occurring after an standard 100-choice interaction with the IGT; Stocco et al., 2009), individuals can still make their decisions based on representations they have previously acquired through cognitive and affective processes (e.g., Stocco et al., 2009).

In the present review, based on this dual-process model and on recent influential theoretical accounts (Hofmann and Friese, 2008; Hofmann et al., 2009; Verdejo-Garcia and Bechara, 2009; Stacy and Wiers, 2010; Noël et al., 2013), we argue that PGs' exaggerate the salience associated with gambling cues to the point that these cues literally “hijack” the cognitive and affective reflective processes necessary to choose on the basis of both short-term and long-term outcomes. In other words, the “working hypothesis” here is that the extreme saliency associated with high short-term rewards in PG detrimentally impacts their decision-making profile during the IGT.

## Gambling disorder and IGT performance

There is a convergence in findings from studies examining decision-making using the IGT in PG (see also Table 1). More specifically, abstinent (e.g., Goudriaan et al., 2005) or non-abstinent (e.g., Power et al., 2012) PG with (e.g., Cavedini et al., 2002) or without co-morbid substance (e.g., Brevers et al., 2012a) abuse seem to display a stubborn preference for disadvantageous deck selection during the IGT, as compared with healthy control participants. Nevertheless, a couple of studies reported non-significant difference between PG and controls on the IGT (Tanabe et al., 2007; Linnet et al., 2011a,b, 2012; De Wilde et al., 2013). This finding could be due to the low sample size of the PG group recruited in these studies (see Table 1). This absence of significant difference might also stem from the heterogeneity of gambling addiction (even if PGs' preferred gambling was not reported in these studies). More specifically, the literature dichotomizes gambling activities into non-strategic (e.g., slot machines games) and strategic (e.g., poker) gambling (e.g., Potenza, 2001; Grant et al., 2012). Strategic gambling conceivably involves different cognitive demands than non-strategic gambling. Poker, for example, in addition to involve “hot” emotional self-regulation (bluffing, regulation of loss-induced frustration; Palomäki et al., 2013), requires “cool” executive processes such as, working memory and mental flexibility (e.g., keeping track of cards played to determine odds of receiving a certain card). Hence, one may infer that strategic gamblers differ from non-strategic gamblers on several neuropsychological processes. Grant et al. (2012) have recently examined this possibility but did not report any difference between strategic (e.g., poker, sports betting, stock market) and non-strategic gamblers (e.g., slots, roulette) with regard to their ability to shift between multiple tasks (i.e., set-shifting) and to inhibit a prepotent motor response. With regard to the IGT, Goudriaan et al. (2005) found a difference in decision-making strategies between slot machine gamblers and casino gamblers (engaged mainly in strategic card games), with the former performing worse than the latter, and the latter not different from their controls.

In light of the limited research, further studies are needed to explore the multiple aspects of “hot” and “cool” EFs in strategic and non-strategic PG. Moreover, the use of complementary profile analyses may bring important information with regard to the multifaceted aspect of the gambling dependence state. For instance, despite a significant between-group difference, up to 30% of healthy controls have been reported to exhibit poor performance on the IGT (Li et al., 2010) and normal performance has also been observed among PGs (Álvarez-Moya et al., 2011). In addition, Peterson et al. (2010) observed that, in both PG and controls, highly sensation-seeking subjects had a significant increase in neural activity in a brain region that receives dopamine projections, i.e., in the ventral striatum (a brain area involved in the anticipation of monetary rewards; Knutson et al., 2003) during the IGT. As a whole, these results support the view that gambling disorder is a multifaceted psychopathological state and that PG may be clustered into distinct subgroups (e.g., high sensation-seeking PG vs. low sensation-seeking PG; Peterson et al., 2010) in future IGT studies.

## Hyperactivity of impulsive processes toward gambling-related cues in PG

The amygdala-striatal “*impulsive*” system has been argued to be responsible for the transfer of reward seeking from controlled to automatic and habitual behaviors (Everitt et al., 1999; Everitt and Robbins, 2005). Those incentive automatic/habitual behaviors are assumed to emerge from the activation of certain associative clusters in long-term memory by perceptual (e.g., words, images, video) or imagined stimulus input (Strack and Deutsch, 2004). These associations are created and strengthened gradually through classical conditioning processes, that is, by the learning history of temporal or spatial coactivation between external stimuli and affective reactions (Hofmann et al., 2008, 2009). These associative clusters endow the organism the ability to evaluate and respond to the environment quickly in accordance with one's current needs and previous learning experiences (Hofmann et al., 2008, 2009). When, for example, the gambler encounters gambling-related cues, the “gambling cluster” may get reactivated, which will automatically trigger a corresponding impulse, consisting of a positive incentive value attributed to gambling and a corresponding behavioral schema to approach it (Stacy and Wiers, 2010). In other words, repeated and marked “high” throughout the repetition of gambling experiences, learned associations between gambling-rewards hedonic effects and stimuli in the environment endow these gambling-related cues with the ability to directly access the mental representations associated with the action of gambling and, like gambling itself, make them attractive (Hofmann et al., 2009). As a result, gambling-related cues may be flagged as salient and automatically trigger motivation-relevant associative memories (i.e., implicit association) and may also grab the addicts' attention (i.e., attentional bias) (Stacy and Wiers, 2010).

So far, two studies (Yi and Kanetkar, 2010; Brevers et al., 2013a) have directly investigated implicit association (i.e., spontaneous associations between addiction related cues and affective, arousal, motivational representation in memory, which are independent of, or not available to, conscious awareness; Greenwald and Banaji, 1995) toward gambling-related cues in PG. More specifically, these studies showed that PG exhibited positive, but not negative implicit associations toward gambling cues on the well-known Implicit Association Task (Greenwald et al., 1998). Several studies have also emphasized the presence of attentional bias for gambling related stimuli in PG. For instance, two recent studies (Brevers et al., 2011a,b) found that PG exhibit attentional bias (i.e., a modified attentional processing for addiction-relevant stimuli; Franken, 2003) toward gambling-related cues at early stage of attentional processing (e.g., attentional encoding; initial orientation of attention), which depends essentially on automatic-habit processes (Browning et al., 2010; Cisler and Koster, 2010). Other evidence for the presence of attentional bias in problem gambling comes from Zack and Poulos (2004), who investigated whether gambling-like drugs could prime the addiction-related implicit cognition network. More specifically, these authors observed that, during a rapid reading task in which target words were degraded with asterisks (e.g., w^*^a^*^g^*^e^*^r), a dopamine agonist amphetamine (dopamine is a neurotransmitter that plays a major role in reward-driven learning for every type of rewards) heightened PG readiness to read gambling-related words while concurrently slowing reading speed of neutral words (Zack and Poulos, 2004). In addition, Zack and Poulos (2004) showed that the dopamine agonist enhanced self-reported motivation to gamble in PG. These results suggest that activation of the mesolimbic dopamine system gives rise to an incentive “seeking” state, which also involves the collateral suppression of alternative motivations.

Enhanced saliency for gambling-related cues in problem gamblers has also been highlighted by functional magnetic resonance imaging (fMRI) research on cue reactivity (Crockford et al., 2005; Goudriaan et al., 2010; but see Potenza et al., 2003). For instance, Goudriaan et al. (2010) observed that, while viewing gambling-related pictures, PG exhibited higher brain activation than controls in areas involved in the reactivity to emotional information (i.e., the amygdala; Gallagher and Chiba, 1996), in the formation of interoceptive representation (the insular cortex; Craig, 2009), and in the regulation of emotional input (i.e., the VMPC; Rolls and Grabenhorst, 2008). In addition, these authors observed that subjective ratings of craving in PG correlated positively with brain activation in the VMPC and in the insular cortex. These results are important because they suggest that the perception of gambling cues in PG trigger gambling urge, which encompass brain areas involved in impulsive emotional processes (the amygdala, the insula), as well as “hot” EFs (i.e., VMPC activation).

## Hyperactive impulsive processes and impaired IGT performance in PG

Findings depicted in the previous section suggest that problem gambling is underlined by powerful impulsive motivational-habit machinery directed at gambling-related cues, which could possibly interfere or “hijack” the top-down reflective mechanisms necessary for triggering alarming signals about future outcomes. Therefore, one can assume that similar processes may bias PGs' decision-making during the IGT toward options featuring high, short-term rewards.

Findings from brain-imaging studies on the IGT in gambling disorder are in line with this assumption. Indeed, recent positron emission tomography (PET) studies found that, in contrast to their comparison controls, disadvantageous performance on the IGT was associated with dopaminergic release in the ventral striatum in PG (Linnet et al., 2010, 2011a). More specifically, whereas in healthy controls dopamine is released in response to advantageous deck choices, in PG, disadvantageous deck selections (Linnet et al., 2010, 2011a) and subjective excitement (Linnet et al., 2011b) are higher in response to dopamine release. Using fMRI technique, Power et al. (2012) have observed that, during high-risk choice in the IGT, PG exhibited increased activation in regions encompassing the extended reward pathway, including brain areas involved in the integration of emotional and cognitive input (i.e., the orbitofrontal cortex, OFC; Rolls and Grabenhorst, 2008), involved in the reactivity to emotional information (i.e., the amygdala) and in short-term reward-based behavioral learning (i.e., caudate nucleus; Haruno and Kawato, 2006). However, in another fMRI study, Tanabe et al. (2007) observed a diminished VMPFC activation during the IGT in SD individuals and also individuals who are SD and PG (SDPG). Since these studies did not focus on pure PG, it is important to caution that the observed diminished VMPFC activation might not be due to gambling addiction alone, but rather to repeated ingestions of exogenous substance that cause harmful effects in the brain

A main limitation of these brain-imaging studies (both PET and fMRI) is that components of decision-making during the IGT have not been broken down into more specific processes that allow a better evaluation of the differential brain activation associated with different steps of decision-making. More specifically, it is unclear whether enhanced impulsive processes toward disadvantageous deck selection is related to outcome anticipation (i.e., when the subject is pondering potential options before making a decision; Cohen and Ranganath, 2005), outcome expectation (i.e., the subject has made a decision and waits the outcome; van Holst et al., 2012) or outcome processing (i.e., the subject receive a feedback on the chosen option). This issue have been recently addressed by two fMRI studies which have investigated neural activation associated with the outcome anticipation (Miedl et al., 2010) and expectation (van Holst et al., 2012) phases of gambling-related decision-making in PG. Specifically, Miedl et al. (2010) observed that, before taking high-risk decisions in a quasi-realistic blackjack scenario, PG exhibited enhanced brain responses in the inferior OFC and in the medial pulvinar nucleus (the pulvinar is a relay thalamic nucleus that receives interoceptive input and in turn projects to the insula, all of which are brain areas associated with impulsive urges; Sewards and Sewards, 2003), whereas controls showed a significant signal increase in low-risk conditions, which might reflect a cue-induced signal increase for high-risk situations in PG (Miedl et al., 2010). With regard to outcome expectation, van Holst et al. (2012) showed that, compared with their controls, PG exhibited higher activity in the ventral striatum and the OFC during the expectation of gambling-related outcome.

Altogether, findings from brain-imaging studies suggest that disadvantageous decision-making during the IGT (or during others situations of monetary gambling) in PG may be due to their hypersensitivity, or exaggerated salience, to immediate and larger monetary rewards. In other words, in PG, the need to make a gambling-related choice (i.e., disadvantageous decks during the IGT) could be so high that it could literally “hijack” the “hot” reflective resources (evidenced through OFC activation) toward short-term gratification. Nevertheless, it is noteworthy that these brain-imaging findings are in apparent contradiction with psychophysiological findings from Goudriaan et al. (2006) who observed lowered skin conductance and heart rate responses associated with disadvantageous deck selection in PG, as compared to controls. Indeed, hyperactivity in the fronto-striatal brain reward pathway is typically associated with higher autonomic-arousal responses. For instance, striatal (e.g., Salimpoor et al., 2011) and VMPC (e.g., Wong et al., 2007) activations have been associated with greater heart rate and skin conductance response. Hence, further studies are needed to implement a careful online measurement of autonomic arousal during fMRI scanning (for a review on how integrating fMRI with psychophysiological measurements during the IGT, see Wong et al., 2011), which would complement fMRI findings in providing a more comprehensive understanding on the physiological and neural mechanisms of impaired decision-making in PG. Moreover, additional studies are needed in order to examine the association between IGT and other indexes of “hot” executive processes, that is, processes involved in the regulation of short-term reward in PG. One option would be to examine the association between the IGT and the delay discounting task (DDT; Madden et al., 1997). In this task, individuals are to choose between smaller immediate rewards and larger, delayed rewards (e.g., $9 immediately vs. $15 in 1 week). Several studies showed that, as compared with their controls, PG exhibited a higher intolerance to delayed gratification on the DDT (e.g., Brevers et al., 2012b). Moreover, evidence suggests that the OFC play an important role in the capacity to delay reward on the DDT (e.g., Rogers et al., 1999; Rahman et al., 2001; Krawczyk, 2002). In addition, Monterosso et al. (2001) found that performance on the IGT was significantly correlated with performance on the DDT in a group of cocaine-dependent individuals. These findings suggest that the IGT and the DDT tap similar affective decision-making processes.

Importantly, it appears that there is no association between impairments in “cool” executive functioning and IGT performance in PG (for a review on “cool” EFs impairments in PG, see Goudriaan et al., 2004; van Holst et al., 2010). Roca et al. (2008) examined IGT performance and prepotent motor response inhibition (i.e., the ability to deliberately suppress dominant, automatic responses that are no longer relevant or required) in 11 PG and 11 controls. These authors showed that PG performed worse than controls on the IGT, and they had a poorer ability to inhibit prepotent responses as assessed with a GO/NO-GO task. However, there was no significant correlation between GO/NO-GO commission errors and overall IGT performance. More recently, based on some evidence supporting that inhibitory processes may be more important during the latter half of the IGT (Noël et al., 2007; see also BOX 1 for a discussion on the association between “cool” EFs and latter stages of the IGT), Kertzman et al. (2011) examined the association between IGT and prepotent motor response inhibition (GO/NO-GO and Stroop task) as a function of early (trials 1–40) and latter (trials 41–100) stages of IGT performance. However, as in Roca et al. (2008), Kertzman et al. (2011) found no significant relationship between impaired response inhibition in PG and their disadvantageous decision-making during the latter stages of the IGT. According to these authors, the fact that impaired IGT performance in PGs was not a direct result of their impaired inhibition functioning may be an expression of more general executive functioning deficits (e.g., working memory, cognitive flexibility). However, this assumption is not congruent with findings from a recent study by Brevers et al. (2012a) which highlighted that PGs' impaired performance on dual tasking (a main central executive components of working memory) was not correlated with their lowered IGT performance, at either the early or the latter stages of IGT. These findings suggest that impaired IGT performance in PG is independent from their deficit in “cool” executive processes. To a broader extent, these results are in line with theoretical accounts which advance that before elaborate decontextualized problem-solving abilities and other related cognitive skills can begin to be enacted, the ability to control emotional reactions and inhibit basic behavioral impulses is required first (Barkley, 1997; Sonuga-Barke et al., 2002; Giancola et al., 2012). Put differently, the “hijack” of impulsive incentive process on the “hot” reflective resources would hamper further elaborated decontextualized problem-solving abilities (i.e., “cool” executive processes). Further studies are needed in order to confirm that impaired “cool” executive processes do not impact PGs' IGT performance. One option would be to increase the number of IGT trials (e.g., from 100–120) and to examine the association between these later trials and performance on tasks estimating “cool” EFs. Indeed, the impact of “cool” is higher during the later trials of the IGT (see BOX 1). Another option would be to use the IGT with the reversal contingencies condition (Fellows and Farah, 2005). In this task the initial reward/punishment schedule are rearranged such that the two disadvantageous decks no longer had an initial advantage in the opening trials. Hence, if PGs obtain same performances as those of healthy controls, it would suggest that it is a difficulty in reversing early learning that is underpinning the behavioral profile of PG on the IGT (Dunn et al., 2006).

Box 1 **The impact of “cool” EFs during the IGT**

The IGT has been shown to tap into “hot” EFs, that is, aspects of decision-making that are influenced by affect and emotion (Bechara, 2004). Specifically, Bechara and colleagues have demonstrated that, whereas healthy controls learn to avoid the disadvantageous decks, patients with damage to VMPFC continue to choose from these disadvantageous decks (e.g., Bechara et al., 1994, 1997, 2000). Nevertheless, several recent findings suggest that not all aspects of the IGT are equal at detecting “hot” decision-making processes. Consistent with this view, performances on working memory (Brevers et al., 2012a), dominant response inhibition (Noël et al., 2007) and cognitive flexibility (Brand et al., 2007; Iudicello et al., 2013) have been associated with performance of healthy controls on the latter stages of the IGT. Hence, these results suggest that “cool” executive processes may be involved in the latter trials of the IGT.

One explanation for these findings is that, across trials, the IGT may vary according to its level of uncertainty (Brand et al., 2006). More specifically, selections during the last block of trials may be referred as decision-making under risk (i.e., situations of decision-making in which probabilities of reward and loss are known) because participants should have experienced the different win/loss contingencies enough to know which decks are risky and which are not. By contrast, because there has not been time for a participant to experience any of the win/loss contingencies during early deck choices, the first blocks of the IGT refer to decision-making under ambiguity (i.e., situations of decision-making in which probabilities of reward and loss are unknown).

Several theoretical accounts advance that processes underlying decision-making may depend upon the degree of uncertainty and the amount of information offered to the decision-maker (e.g., Brand et al., 2006; Krain et al., 2006). More specifically, because it does not offers explicit rules for possible outcomes or probabilities, decision-making under ambiguity has to be made via the reactivation of emotions associated with similar previous experiences (i.e., “hot” executive processes; Brand et al., 2006; Krain et al., 2006). By contrast, decision-making a decision under risk, which offers explicit rules for reinforcement and punishment, would involve both the integration of pre-choice emotional processes and rational analytical system aspects (i.e., “cool” executive processing; Brand et al., 2006; Krain et al., 2006). In other words, deteriorations in “hot” and “cool” executive functions could alter differently decision-making under risk and decision-making under ambiguity. For instance, Brand et al. (2007) observed that individuals with lowered “cool” executive functioning (i.e., concept formation, shifting between multiple tasks, and dominant response inhibition) but with intact “hot” executive processing (i.e., pre-choice emotional activation reactivity associated with an advantageous decision-making profile) exhibited less disadvantageous choices in situations of decision-making under ambiguity as compared with situations of decision-making under risk. By contrast, Brand et al. (2007) also found that individuals with selective deficits in pre-choice emotional activation but with intact “cool” executive functioning exhibited disadvantageous choices in decision-making under risk and under ambiguity. Additional studies have shown that advantageous decision-making under risk, but not under ambiguity, is associated with efficient “cool” executive processing (i.e., calculative strategies; Brand, 2008; Brand et al., 2009). Moreover, advantageous decision-making under risk (Starcke et al., 2011), but not under ambiguity (Turnbull et al., 2005), is lowered when subjects have to take a decision while concurrently performing a secondary task (i.e., random number generation), which are known to load “cool” executive resources (Baddeley and Della Sala, 1996).

## Gambling disorder and post-decision appraisals during the IGT

Throughout this paper, we have seen that PG exhibited poor deck selection during the IGT. But how do they react to the consequences of their choice? More specifically, are PG impaired in their ability to react to loss and reward during the IGT? Goudriaan et al. (2006) have demonstrated that PGs' heart rate decreased after choosing from either the good or bad decks, whereas the heart rate of their controls decreased after disadvantageous choices, but increased after advantageous choices. These findings indicate that, as compared to controls, PG exhibit decreased reactivity to rewards and losses during the IGT. Furthermore, in another study, Goudriaan et al. (2005) observed that, compared to controls, PG displayed a higher response speed and lower response shifting after rewards and net losses. Taken together, findings from Goudriaan et al. (2005, 2006) are consistent with several brain imaging studies that observed a reduction of cerebral activity for the processing of rewards and losses in PG during monetary gambling task (Reuter et al., 2005; de Ruiter et al., 2009). Nevertheless, Oberg et al. (2011) have recently observed that disadvantageous IGT deck selection in PG was associated with a hypersensitive neural response at a very early (i.e., 185 ms) post-feedback latency (i.e., the MedioFrontal Negativity, which is involved in the early, rapid positive vs. negative appraisal of feedback; Yeung et al., 2004), but lower neural activity at a later phase (i.e., 300 ms) of feedback processing (i.e., the P300 Theta Amplitude which reflects a later, attention-sensitive, more elaborated appraisal of outcome evaluation; Sato et al., 2005). Hence, these results indicate that, although PG may exhibit a blunted absolute response to outcome signals in general, the neurobiology of feedback processing in problem gambling is probably more complex. Noteworthy, mean age of PG participants recruited by Oberg et al. (2011) was 23 and their scores of problem gambling severity were relatively low. Hence, in Oberg et al. (2011), PGs' hypersensitivity to reward at early post-feedback latency might be due to the fact that they were at an early-stage of problem gambling and had not yet suffered the long-term consequences of excessive gambling (e.g., tolerance to money reward). Further longitudinal investigations would be helpful in evaluating the potential use of Oberg et al. (2011) findings as an early indicator of predisposition to gambling or other addictive behaviors.

As a whole, these results indicate that, throughout the repetition of gambling behaviors, PG acquire an extensive experience in making complex financial decisions involving variable wins, losses and probabilities. Thus, while gambling disorder does not entail exogenous drug administration, neural systems that process rewards may nonetheless undergo neuroadaptive change as the gambler experiences a chronic regime of winning and losing, coupled with the changes in arousal that are induced by those events. Because of this tolerance, problem gamblers may start to act out more frequently and, sometimes, in more dangerous ways by often gambling with greater and greater stakes toward options featuring high but uncertain rewards.

Are PG also impaired in their ability to assess the quality of their already poor decisions? In other words, is there a dissociation between PGs' subjective evaluation of IGT performance and their actual performance (i.e., metacognitive ability)? Such impairment of metacognitive capacity in individuals suffering from addiction may be reflected in one of the most common observation from the clinic of addiction, that is, impairment in recognition of the severity of the disorder by the addict (i.e., lack of insight; Goldstein et al., 2009). For instance, only 4.5% of the 21.1 million persons classified as needing (but not receiving) substance use treatment reported a perceived need for therapy (SAMHSA, 2007). Hence, when metacognitive judgment becomes exceedingly disrupted, the repetition of addiction-related behaviors may be heightened by the underestimation of addiction severity.

Metacognitive judgment during the IGT has been recently examined in PG by Brevers et al. (2013b). These authors examined metacognitive capacities in PG by asking participants to wager on their own decisions after each choice during the IGT (i.e., IGT with post-decision wagering; Persaud et al., 2007). These authors observed that, unlike controls, PG participants tend to wager high while performing poorly on the IGT. This result suggests that PG exhibited impairments not only in their ability to correctly assess risk in situations that involve ambiguity, but also in their ability to correctly express metacognitive judgments about their own performance. That is, PG not only perform poorly, but they also erroneously estimate that their performance is much better than it actually is. In line with these findings, Goudriaan et al. (2005) showed that PG exhibited lower IGT conceptual knowledge than their controls when they were asked to indicate which decks were advantageous or disadvantageous. Interestingly, in another recent study, Brevers et al. (2013c) showed that PG were also impaired in their capacity to evaluate accurately the quality of their decisions during a non-gambling task in which the quality of choice remains uncertain throughout the task (i.e., an artificial grammar-learning paradigm). After each trial of this task, participants had to indicate how confident they were in their grammaticality judgments. Results showed that, by contrast with their controls, there was no correlation between PGs' grammaticality judgments and their level of confidence, which suggests a disconnection between performance and confidence in PG. To a broader extent, these findings indicate that PG are impaired in their metacognitive abilities on a non-gambling task, which suggests that gambling disorder is associated with poor insight as a general factor.

Future studies are needed to confirm this assumption. The use of functional neuroimaging studies, which could probe the neural basis of these deficits, is one option. Indeed, a recent investigation showed that the prefrontal cortex, and especially areas involved in “cool” EFs, such as the dorsolateral prefrontal cortex, are activated while subjects report metacognitive judgment on their performance during “neutral” situations of decision-making. For instance, Del Cul et al. (2009) have demonstrated that prefrontal lesions could affect subjective reports of visual experience more than visual task performance. Moreover, Slachevsky et al. (2001, 2003) have shown that lesion affecting the prefrontal cortex also affects awareness as well as the monitoring of actions or sensory-motor readjustments. Other studies showed that bilaterally-depressed activity in the dorsolateral prefrontal cortex, through transcranial magnetic stimulation, can affect metacognition but not task performance during a visual discrimination task (Turatto et al., 2004; Rounis et al., 2010).

## Summary

**Figure 1:**
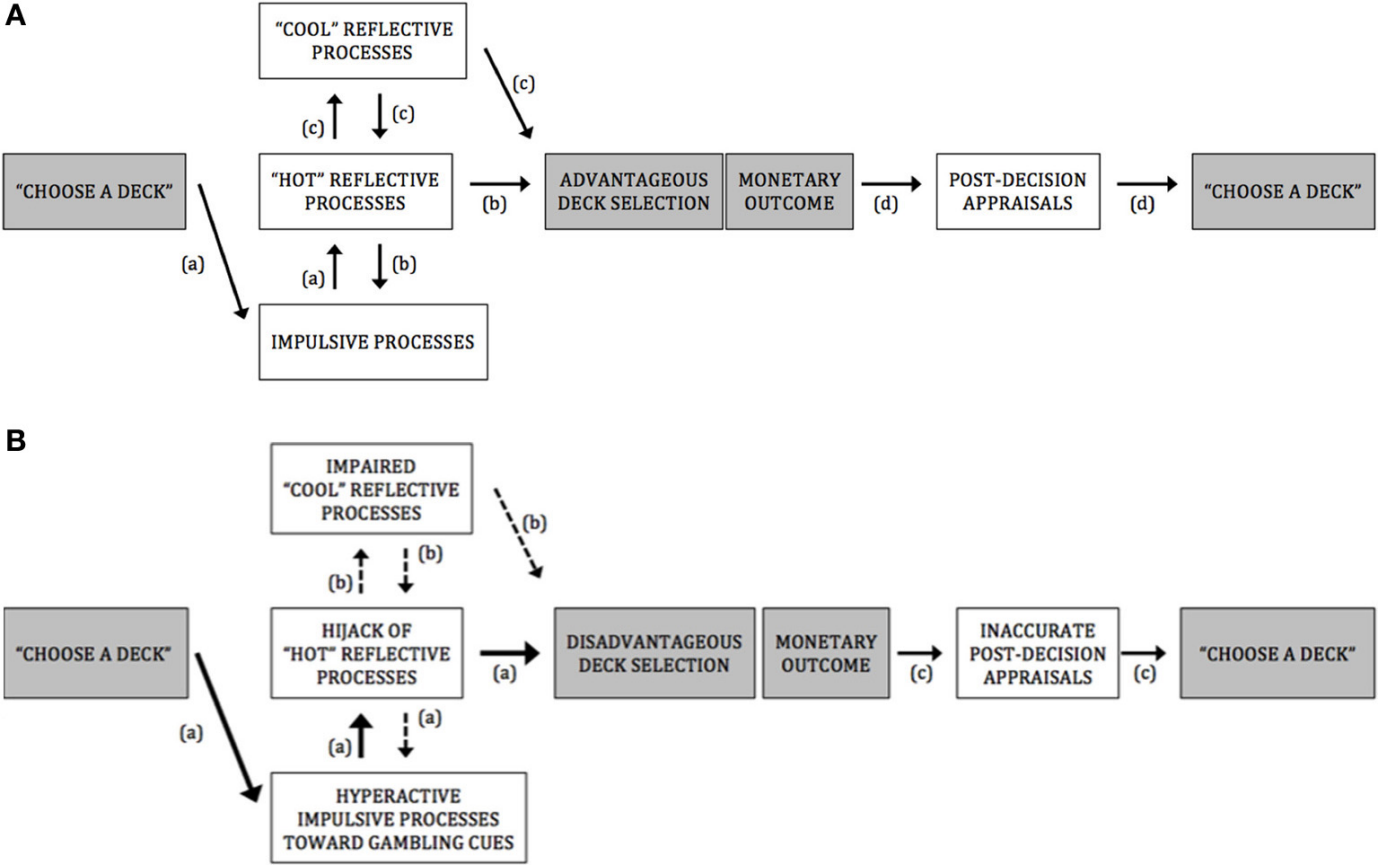
**(A)** A framework for advantageous deck selection in healthy controls. *Pathway (a)*: Impulsive motivational processes directed at options featuring short-term salient rewards. *Pathway (b)*: The moderation of impulsive processes by “hot” reflective processes involved in the reduction of impulsive-incentive reactions and in the ability to anticipate the potential outcomes of a given decision on an emotional basis. *Pathway (c)*: The ability to control emotional reactions and inhibit basic behavioral impulses by “hot” executive/reflective functions allows rational and cognitive determinations of risks and benefits associated with options (only during the last trials of the IGT, that is, when participants have experienced the different winl/loss contingencies enough and become aware of which decks are more at risk than others), which further reinforce the efficiency of reward anticipation processes (e.g., to weigh short-term gains against long-term losses on both emotional and rational bases). *Pathway (d)*: Adequate sensitivity to loss and reward and accurate assessment of the quality of the decision, which would bias advantageously forthcoming deck selections. **(B)** A framework for disadvantageous deck selection in pathological gamblers. *Pathway (a)*: Hyperactive impulsive motivational processes directed at options featuring high, short-term rewards (as evidenced with attentional bias and implicit association toward gambling-related cues in PG; *see* Hyperactivity of impulsive processes toward gambling-related cues in PG). These impulsive processes could possibly interfere with or “hijack” the top-down “hot” reflective mechanisms necessary for triggering alarming signals about futures outcomes (as evidenced by fMRI studies which showed that, during disadvantageous lGT choice or during gambling·-related choice, PG exhibit increased activation in brain regions encompassing both impulsive-amygdala, ventral striatum, caudate nucleus, medial pulvinar nucleus - and “hot” reflective·- orbitofrontal cortex - processes; *see* Hyperactive impulsive processes and impaired IGT performance in PG). As a result, disadvantageous deck options may be flagged as salient and preferred to advantageous decks. *Pathway (b)*: The “hijack” by impulsive incentive processes of the “hot” reflective resources would hamper further elaborated decontextualized problem-solving abilities (suggested by the absence of correlation between PGs' impairments in “cool” executive functioning and their lowered IGT performances, at either the early or the latter stages of IGT; *see* Hyperactive impulsive processes and impaired IGT performance in PG). *Pathway (c)*: Hyposensitivity to loss and reward in PG (as evidenced by fMRI studies which observed a diminished ventral striatal response in PG after receiving monetary rewards and losses; *see* Gambling disorder and post-decision appraisals during the IGT) and failure at correctly assessing the quality of their already poor decision (evidenced by studies which observed a dissociation between PGs' subjective assessment of performance and objective performance; *see* Gambling disorder and post-decision appraisals during the IGT). As a result, PG might fail at properly integrate the outcomes of their actions over time, which could lead them to persist in taking high-risk choices, despite suffering large losses.

PG display a stubborn preference for disadvantageous deck selection throughout the IGT, which suggest that they are hampered in their ability to resist short-term high and uncertain rewards. In this paper, based on dual-process model of willpower (e.g., Bechara, 2005; Everitt and Robbins, 2005; Redish et al., 2008), and on recent influential theoretical accounts (Hofmann et al., 2008, 2009; Verdejo-Garcia and Bechara, 2009; Stacy and Wiers, 2010; Noël et al., 2013), we advanced the view that this inability to forgo short-term benefits for long-term benefits may be underlined by an exaggerated response to cues predicting immediate and large monetary rewards (see Figure 1 for a framework summarizing processes underlying A. advantageous deck selection in healthy controls and B. disadvantageous deck selection in pathological gamblers).

We first reviewed findings showing that gambling-related cues automatically trigger PGs' motivation-relevant associative memories (Yi and Kanetkar, 2010; Brevers et al., 2013a) and grab the addicts' attention (e.g., Brevers et al., 2011a,b). In addition, findings from cue reactivity studies suggest that scores of subjective craving correlated positively with PGs' brain activation in areas involved in impulsive/automatic emotional processes (i.e., the amygdala, the insula) but also in “hot” EFs (i.e., the VMPC) (Crockford et al., 2005; Goudriaan et al., 2010). These results suggest that gambling disorder is underlined by powerful impulsive motivational-habit machinery directed at gambling-related cues, which could possibly bias PGs' decision-making during the IGT toward option featuring high, short-term rewards.

Accordingly, we then focused on studies investigating processes involved in PGs' impaired IGT performance. PET studies highlighted that disadvantageous performance on the IGT was associated with dopaminergic release in the ventral striatum in PG (Linnet et al., 2010, 2011a,b, 2012). Moreover, fMRI findings (Power et al., 2012) observed that, in line with cue-reactivity studies (e.g., Goudriaan et al., 2010), high-risk choice during the IGT in PG was underlined by an increased neural activation in regions involved in the reactivity to emotional information (i.e., the amygdala), in short-term reward-based behavioral learning (i.e., the caudate nucleus), and in the integration of emotional and cognitive input (i.e., the OFC). In other words, these results suggest that the incentive-salience associated with gambling-related choice (i.e., disadvantageous decks selection during the IGT) in PG is so high that it could literally “hijack” the “hot” reflective resources toward short-term gratifications. In addition, it appears that PGs' impairments in “cool” executive processes, including working memory (Brevers et al., 2012a) and response inhibition (Roca et al., 2008; Kertzman et al., 2011), are not associated with their disadvantageous decks selection, at both early (e.g., trials 1–40) or late (e.g., trials 41–100) stages of IGT performance. These findings suggest that PGs' impaired IGT performances are not due to their lower level of “cool” EFs.

In the last part of this paper, we highlighted the issue that gambling disorder might also be associated with a diminished feedback reactivity during the IGT. In addition, recent findings suggest that PG not only perform poorly on the IGT, but they also erroneously estimate that their performance is much better than it actually is (Brevers et al., 2013b). These findings on feedback reactivity and metacognitive capacity imply that PG might fail at properly integrating the outcomes of their actions over time in order to form a global impression of the trade-offs between risk and reward, which could lead them to persist in taking high-risk choices, despite suffering large losses.

## Future studies

As suggested throughout this paper, additional studies are needed in order to further examine the processes associated with impaired IGT performance in PG. For instance, future studies should examine the association between IGT and other tasks estimating “hot” executive processes, such as the delayed discounting task (e.g., Hongwanishkul et al., 2005). Moreover, additional fMRI studies are also needed in order to better evaluate differential brain activation as it relates to different phases of decision-making during the IGT (i.e., outcome anticipation, outcome expectation, and outcome processing). It should also be useful to implement a careful online measurement of autonomic arousal during the fMRI scanning, which would complement fMRI findings in providing a more comprehensive understanding on the physiological and neural mechanisms underlying impaired decision-making in PG (e.g., Wong et al., 2011). Further studies are also needed in order to confirm that impaired “cool” executive processes do not impact PGs' IGT performance, by using for instance, the IGT with the reversal contingencies condition (Fellows and Farah, 2005) or by increasing the number of IGT trials (because the impact of “cool” is higher during the later trials of the IGT). Finally, future studies should also assess pre- and post-IGT gambling-related craving in PG. Indeed, recent theoretical accounts argue that the subjective experience of urge and craving may increase the drive and motivation to gamble (and to choose decks featuring high reward but higher losses during the IGT) in PG by sensitizing or exacerbating the activity of the habit/impulsive system, and by subverting attention, reasoning, planning, and decision-making processes to seek and access gambling (Verdejo-Garcia and Bechara, 2009; Sutherland et al., 2012; Noël et al., 2013).

## Conclusion

In conclusion, because it mimics both real life and gambling-related decision-making situations, the IGT may be the most ecologically valid estimation of decision-making impairments in PG. Accordingly, through the use of this task, studies on gambling addiction have yielded a consistent view of disadvantageous decision-making in PG. In this review, we advanced that this aberrant profile of decision-making may be underlined by a hyperactivity of impulsive processes toward high-uncertain rewards, which can interfere with “hot” and “cool” reflective resources necessary for self-regulation. Nevertheless, much as to be done as it remains unclear on how these processes contribute specifically to the aberrant choice profile displayed by PG on the IGT.

### Conflict of interest statement

The authors declare that the research was conducted in the absence of any commercial or financial relationships that could be construed as a potential conflict of interest.

## References

[B1] Abbott M. W. Volberg R. A. (1995). The New Zealand national survey of problem and pathological gambling. J. Gambl. Stud. 12, 143–160 10.1007/BF01539171 24233913

[B2] American Psychiatric Association. (2013). Diagnostic and Statistical Manual of Mental Disorders, 5th Edn. Arlington, VA: American Psychiatric Publishing

[B3] Álvarez-Moya E. M. Ochoa C. Jiménez-Murcia S. Aymamí M. N. Gómez-Peña M. Fernández-Aranda F. (2011). Effect of executive functioning, decision-making and self-reported impulsivity on the treatment outcome of gambling disorder. J. Psychiatry Neurosci. 36, 165–175 10.1503/jpn.090095 21138656 PMC3080512

[B4] Baddeley A. Della Sala S. (1996). Working memory and executive control. Philos. Trans. R. Soc. Lond.B Biol. Sci. 351, 1397–1403 discussion: 1403–1404. 10.1098/rstb.1996.0123 8941951

[B5] Barkley R. (1997). Attention deficit/hyperactivity disorder, self-regulation, and time: towards a more comprehensive theory. J. Dev. Behav. Pediatr. 18, 271–279 10.1097/00004703-199708000-00009 9276836

[B6] Bechara A. (2004). The role of emotion in decision-making: evidence from neurological patients with orbitofrontal damage. Brain Cogn. 55, 30–40 10.1016/j.bandc.2003.04.001 15134841

[B7] Bechara A. (2005). Decision-making, impulse control, and loss of willpower to resist drugs: a neurocognitive perspective. Nat. Neurosci. 8, 1458–1463 10.1038/nn1584 16251988

[B8] Bechara A. (2007). Iowa Gambling Task (IGT) Professional Manual. Lutz, FL: Psychological Assessment Resources

[B9] Bechara A. Damasio A. R. Damasio H. Anderson S. W. (1994). Insensitivity to future consequences following damage to human prefrontal cortex. Cognition 50, 7–15 10.1016/0010-0277(94)90018-3 8039375

[B10] Bechara A. Damasio H. Tranel D. Damasio A. R. (1997). Deciding advantageously before knowing the advantageous strategy. Science 275, 1293–1294 10.1126/science.275.5304.1293 9036851

[B11] Bechara A. Dolan S. Denburg N. Hindes A. Anderson S. W. Nathan P. E. (2001). Decision-making deficits, linked to a dysfunctional ventromedial prefrontal cortex, revealed in alcohol and stimulant abusers. Neuropsychologia 39, 376–389 10.1016/S0028-3932(00)00136-6 11164876

[B12] Bechara A. Tranel D. Damasio H. (2000). Characterization of the decision-making impairment of patients with bilateral lesions of the ventromedial prefrontal cortex. Brain 123, 2189–2202 10.1093/brain/123.11.2189 11050020

[B13] Brand M. (2008). Does the feedback from previous trials influence current decisions? A study on the role of feedback processing in making decisions under explicit risk conditions. J. Neuropsychol. 2, 431–443 10.1348/174866407X220607 19824172

[B14] Brand M. Labudda K. Markowitsch H. J. (2006). Neuropsychological correlates of decision-making in ambiguous and risky situations. Neural Netw. 19, 1266–1276 10.1016/j.neunet.2006.03.001 16942857

[B15] Brand M. Laier C. Pawlikowski M. Markowitsch H. J. (2009). Decision-making with and without feedback: the role of intelligence, strategies, and cognitive styles. J. Clin. Exp. Neuropsychol. 31, 967–981 10.1080/13803390902776860 19358007

[B16] Brand M. Recknor E. C. Grabenhorst F. Bechara A. (2007). Decisions under ambiguity and decisions under risk: correlations with executive functions and comparisons of two different gambling tasks with implicit and explicit rules. J. Clin. Exp. Neuropsychol. 29, 86–99 10.1080/13803390500507196 17162725

[B17] Brevers D. Cleeremans A. Tibboel H. Bechara A. Kornreich C. Verbanck P. (2011a). Reduced attentional blink for gambling-related stimuli in problem gamblers. J. Behav. Ther. Exp. Psychiatry 42, 265–269 10.1016/j.jbtep.2011.01.005 21349247

[B18] Brevers D. Cleeremans A. Bechara A. Laloyaux C. Kornreich C. Verbanck P. (2011b). Time course of attentional bias for gambling information in problem gambling. Psychol. Addict. Behav. 25, 675–682 10.1037/a0024201 21688874 PMC3792789

[B19] Brevers D. Cleeremans A. Goudriaan A. E. Bechara A. Kornreich C. Verbanck P. (2012a). Decision making under ambiguity but not under risk is related to problem gambling severity. Psychiatry Res. 200, 568–574 10.1016/j.psychres.2012.03.053 22521232

[B20] Brevers D. Cleeremans A. Verbruggen F. Bechara A. Kornreich C. Verbanck P. (2012b). Impulsive action but impulsive choice determines problem gambling severity. PLoS ONE 7:e50647 10.1371/journal.pone.0050647 23209796 PMC3507734

[B21] Brevers D. Cleeremans A. Hermant C. Tibboel H. Kornreich C. Verbanck P. (2013a). Implicit gambling attitudes in problem gamblers: positive but not negative implicit associations. J. Behav. Ther. Exp. Psychiatry 44, 94–97 10.1016/j.jbtep.2012.07.008 22940084

[B22] Brevers D. Cleeremans A. Bechara A. Greisen M. Kornreich C. Verbanck P. (2013b). Impaired self-awareness in pathological gamblers. J. Gambl. Stud. 29, 119–129 10.1007/s10899-012-9292-2 22273773

[B23] Brevers D. Cleeremans A. Bechara A. Kornreich C. Verbanck P. Noël X. (2013c). Impaired metacognitive capacities in problem gamblers. J. Gambl. Stud. [Epub ahead of print]. 10.1007/s10899-010-9348-3 23149513

[B24] Browning M. Holmes E. A. Harmer C. J. (2010). The modification of attentional bias to emotional information: a review of the techniques, mechanisms, and relevance to emotional disorders. Cogn. Affect. Behav. Neurosci. 10, 8–20 10.3758/CABN.10.1.8 20233952

[B25] Cavedini P. Riboldi G. Keller R. D'Annucci A. Bellodi L. (2002). Frontal lobe dysfunction in gambling disorder patients. Biol. Psychiatry 51, 334–341 10.1016/S0006-3223(01)01227-6 11958785

[B26] Cisler J. M. Koster E. H. W. (2010). Mechanisms of attentional biases towards threat in anxiety disorders: an integrative review. Clin. Psychol. Rev. 30, 203–216 10.1016/j.cpr.2009.11.003 20005616 PMC2814889

[B27] Cohen M. X. Ranganath C. (2005). Behavioral and neural predictors of upcoming decisions. Cogn. Affect. Behav. Neurosci. 5, 117–126 10.3758/CABN.5.2.117 16180619

[B28] Craig A. D. (2009). How do you feel–now? The anterior insula and human awareness. Nat. Rev. Neurosci. 10, 59–70 10.1038/nrn2555 19096369

[B29] Crockford D. N. Goodyear B. Edwards J. Quickfall J. elGuebaly N. (2005). Cue-induced brain activity in pathological gamblers. Biol. Psychiatry 58, 787–795 10.1016/j.biopsych.2005.04.037 15993856

[B30] Damasio A. R. (1996). The somatic marker hypothesis and the possible functions of the prefrontal cortex. Philos. Trans. R. Soc. Lond. B Biol. Sci. 351, 1413–1420 10.1098/rstb.1996.0125 8941953

[B31] Del Cul A. Dehaene S. Reyes P. Bravo E. Slachevsky A. (2009). Causal role of prefrontal cortex in the threshold for access to consciousness. Brain 132, 2531–2540 10.1093/brain/awp111 19433438

[B32] de Ruiter M. B. Veltman D. J. Goudriaan A. E. Oosterlaan J. Sjoerds Z. van den Brink W. (2009). Response perseveration and ventral prefrontal sensitivity to reward and punishment in male problem gamblers and smokers. Neuropsychopharmacology 34, 1027–1038 10.1038/npp.2008.175 18830241

[B33] De Wilde B. Goudriaan A. Sabbe B. Hulstijn W. Dom G. (2013). Relapse in pathological gamblers: a pilot study on the predictive value of different impulsivity measures. J Behav. Addict. 2, 23–30 10.1556/JBA.2.2013.1.4 26165768

[B34] Di Chiara G. (1999). Drug addiction as dopamine-dependent associative learning disorder. Eur. J. Pharmacol. 375, 13–30 10.1016/S0014-2999(99)00372-6 10443561

[B34a] Dunn B. D. Dalgleish T. Lawrence A. D. (2006). The somatic marker hypothesis: A critical evaluation. Neurosci. Biobehav. Rev. 30, 239–271 10.1016/j.neubiorev.2005.07.001 16197997

[B35a] Everitt B. J. Parkinson J. A. Olmstead M. C. Arroyo M. Robledo P. Robbins T. W. (1999). Associative processes in addiction and reward: the role of amygdala-ventral striatal subsystems. Ann. N. Y. Acad. Sci. 877, 412–438 10.1111/j.1749-6632.1999.tb09280.x 10415662

[B35] Everitt B. J. Robbins T. W. (2005). Neural systems of reinforcement for drug addiction: from actions to habits to compulsion. Nat. Neurosci. 8, 1481–1489 10.1038/nn1579 16251991

[B36a] Fellows L. K. Farah M. J. (2005). Different underlying impairments in decision-making following ventromedial and dorsolateral frontal lobe damage in humans. Cereb. Cortex 15, 58–63 10.1093/cercor/bhh108 15217900

[B36] Forbush K. T. Shaw M. Graeber M. A. Hovick L. Meyer V. J. Moser D. J. (2008). Neuropsychological characteristics and personality traits in gambling disorder. CNS Spectr. 13, 306–315 18408650 10.1017/s1092852900016424

[B37] Franken I. A. (2003). Drug craving and addiction: integrating psychological and neuropsychopharmacological approaches. Prog. Neuropsychopharmacol. Biol. Psychiatry 27, 563–579 10.1016/S0278-5846(03)00081-2 12787841

[B38] Gallagher M. Chiba A. A. (1996). The amygdala and emotion. Curr. Opin. Neurobiol. 6, 221–227 10.1016/S0959-4388(96)80076-6 8725964

[B39] Giancola P. R. Godlaski A. J. Roth R. M. (2012). Identifying component-processes of executive functioning that serve as risk factors for the alcohol-aggression relation. Psychol. Addict. Behav. 26, 201–211 10.1037/a0025207 21875167 PMC3288328

[B40] Goldstein R. Z. Craig A. D. Bechara A. Garavan H. Childress A. R. Paulus M. P. (2009). The neurocircuitry of impaired insight in drug addiction. Trends Cogn.Sci. 13, 372–380 10.1016/j.tics.2009.06.004 19716751 PMC2844118

[B41] Goudriaan A. E. de Ruiter M. B. van den Brink W. Oosterlaan J. Veltman D. J. (2010). Brain activation patterns associated with cue reactivity and craving in abstinent problem gamblers, heavy smokers and healthy controls: an fMRI study. Addict. Biol. 15, 491–503 10.1111/j.1369-1600.2010.00242.x 20840335 PMC3014110

[B42] Goudriaan A. E. Oosterlaan J. de Beurs E. van den Brink W. (2004). Gambling disorder: a comprehensive review of biobehavioral findings. Neurosci. Biobehav. Rev. 28, 123–141 10.1016/j.neubiorev.2004.03.001 15172761

[B43] Goudriaan A. E. Oosterlaan J. de Beurs E. van den Brink W. (2005). Decision making in gambling disorder: a comparison between pathological gamblers, alcohol dependents, persons with Tourette syndrome, and normal controls. Cogn. Brain Res. 23, 137–151 10.1016/j.cogbrainres.2005.01.017 15795140

[B44] Goudriaan A. E. Oosterlaan J. de Beurs E. van den Brink W. (2006). Psychophysiological determinants and concomitants of deficient decision-making in pathological gamblers. Drug Alcohol Depend. 84, 231–239 10.1016/j.drugalcdep.2006.02.007 16574343

[B45] Grant J. E. Odlaug B. L. Chamberlain S. R. Schreiber L. R. N. (2012). Neurocognitive dysfunction in strategic and non-strategic gamblers. Prog. Neuropsychopharmacol. Biol. Psychiatry 38, 336–340 10.1016/j.pnpbp.2012.05.006 22613186 PMC3389298

[B46] Grant S. Contoreggi C. London E. D. (2000). Drug abusers show impaired performance in a laboratory test of decision making. Neuropsychologia 38, 1180–1187 10.1016/S0028-3932(99)00158-X 10838152

[B47] Greenwald A. G. Banaji M. R. (1995). Implicit social cognition: attitudes, self-esteem, and stereotypes. Psychol. Rev. 102, 4–27 10.1037/0033-295X.102.1.4 7878162

[B48] Greenwald A. G. McGhee D. E. Schwartz J. K. (1998). Measuring individual differences in implicit cognition: the implicit association test. J. Pers. Soc. Psychol. 74, 1464-1480 10.1037/0022-3514.74.6.1464 9654756

[B49a] Haruno M. Kawato M. (2006). Different neural correlates of reward expectation and reward expectation error in the putamen and caudate nucleus during stimulus-action-reward association learning. J. Neurophysiol. 95, 948–959 10.1152/jn.00382.2005 16192338

[B50a] Hofmann W. Friese M. (2008). Impulses got the better of me: alcohol moderates the influence of implicit attitudes toward food cues on eating behavior. J. Abnorm. Psychol. 117, 420–427 10.1037/0021-843X.117.2.420 18489218

[B49] Hofmann W. Friese M. Strack F. (2009). Impulse and self-control from a dual-systems perspective. Perspect. Psychol. Sci. 4, 162–176 10.1111/j.1745-6924.2009.01116.x 26158943

[B50] Hofmann W. Friese M. Wiers R. W. (2008). Impulsive versus reflective influences on health behavior: a theoretical framework and empirical review. Health Psychol. Rev. 2, 111–137 10.1080/17437190802617668

[B51] Hongwanishkul D. Happaney K. R. Lee W. Zelazo P. D. (2005). Hot and cool executive function: age-related changes and individual differences. Dev. Neuropsychol. 28, 617–644 10.1207/s15326942dn2802_4 16144430

[B52] INSERM. (2008). Expertise Collective: Jeux de Hasard et d'argent, Contexte et Addiction [Collective expert report: gambling, context and addiction]. INSERM, Paris.

[B53] Iudicello J. E. Woods S. P. Cattie J. E. Doyle K. Grant I. (2013). Risky decision-making in HIV-associated neurocognitive disorders (HAND). Clin. Neuropsychol. 27, 256–275 10.1080/13854046.2012.740077 23181946 PMC3609907

[B54a] Kerr A. Zelazo P. D. (2004). Development of “hot” executive function: the children's gambling task. Brain Cogn. 55, 148–157 10.1016/S0278-2626(03)00275-6 15134849

[B54] Kertzman S. Lidogoster H. Aizer A. Kotler M. Dannon P. N. (2011). Risk-taking in pathological gamblers is not a result of their impaired inhibition ability. Psychiatry Res. 188, 71–77 10.1016/j.psychres.2011.02.021 21429591

[B55] Knutson B. Fong G. W. Bennett S. M. Adams C. M. Hommer D. (2003). A region of mesial prefrontal cortex tracks monetarily rewarding outcomes: characterization with rapid event-related fMRI. Neuroimage 18, 263–272 10.1016/S1053-8119(02)00057-5 12595181

[B56] Korn D. A. Shaffer H. J. (1999). Gambling and the health of the public: adopting a public health perspective. J. Gambl. Stud. 15, 289–365 10.1023/A:1023005115932 12766466

[B57] Krain A. L. Wilson A. M. Arbuckle R. Castellanos F. X. Milham M. P. (2006). Distinct neural mechanisms of risk and ambiguity: a meta-analysis of decision-making. Neuroimage 32, 477–484 10.1016/j.neuroimage.2006.02.047 16632383

[B58] Krawczyk D. C. (2002). Contributions of the prefrontal cortex to the neural basis of human decision-making. Neurosci. Biobehav. Rev. 26, 631–664 10.1016/S0149-7634(02)00021-0 12479840

[B59] Lakey C. E. Goodie A. S. Campbell W. K. (2007). Frequent card playing and gambling disorder: the utility of the Georgia gambling task and Iowa gambling task for predicting pathology. J. Gambl. Stud. 23, 285–297 10.1007/s10899-006-9034-4 17171543

[B60] Ledgerwood D. M. Orr E. S. Kaploun K. A. Milosevic A. Frisch G. R. Rupcich N. (2012). Executive function in pathological gamblers and healthy controls. J. Gambl. Stud. 28, 89–103 10.1007/s10899-010-9237-6 21253846

[B61] Leeman F. L. Potenza M. N. (2012). Similarities and differences between gambling disorder and substance use disorders: a focus on impulsivity and compulsivity. Psychopharmacology. 219, 469–490 10.1007/s00213-011-2550-7 22057662 PMC3249521

[B62] Li X. Lu Z. L. D'Argembeau A. Ng M. Bechara A. (2010). The Iowa Gambling Task in FMRI images. Hum. Brain Mapp. 31, 410–423 10.1002/hbm.20875 19777556 PMC2826566

[B63] Linnet J. Møller A. Peterson E. Gjedde A. Doudet D. (2011a). Dopamine release in ventral striatum during Iowa Gambling Task performance is associated with increased excitement levels in gambling disorder. Addiction 106, 383–390 10.1111/j.1360-0443.2010.03126.x 20883460

[B64] Linnet J. Møller A. Peterson E. Gjedde A. Doudet D. (2011b). Inverse association between dopaminergic neurotransmission and Iowa Gambling Task performance in pathological gamblers and healthy controls. Scand. J. Psychol. 106, 383–390 10.1111/j.1360-0443.2010.03126.x 20704689

[B65] Linnet J. Mouridsen K. Peterson E. Møller A. Doudet D. J. Gjedde A. (2012). Striatal dopamine release codes uncertainty in gambling disorder. Psychiatry Res. 204, 55–60 10.1016/j.pscychresns.2012.04.012 22889563

[B66] Linnet J. Peterson E. Doudet D. J. Gjedde A. Møller A. (2010). Dopamine release in ventral striatum of pathological gamblers losing money. Acta Psychiatr. Scand. 122, 326–333 10.1111/j.1600-0447.2010.01591.x 20712823

[B67] Linnet J. Rojskjaer S. Nygaard J. Maher B. A. (2006). Episodic chasing in pathological gamblers using the Iowa gambling task. Scand. J. Psychol. 47, 43–49 10.1111/j.1467-9450.2006.00491.x 16433661

[B68] Madden C. J. Petry N. M. Badger G. J. Bickel W. K. (1997). Impulsive and self-control choices in opioid-dependent patients and non-drug using control participants: drug and monetary rewards. Exp. Clin. Psychopharmacol. 5, 256–263 10.1037/1064-1297.5.3.256 9260073

[B69] Manes F. Sahakian B. Clark L. Rogers R. Antoun N. Aitken M. (2002). Decision-making processes following damage to the prefrontal cortex. Brain 125, 624–639 10.1093/brain/awf049 11872618

[B70] Miedl S. F. Fehr T. Meyer G. Herrmann M. (2010). Neurobiological correlates of problem gambling in a quasi-realistic blackjack scenario as revealed by fMRI. Psychiatry Res. Neuroimaging 181, 165–173 10.1016/j.pscychresns.2009.11.008 20138482

[B71] Monterosso J. Ehrman R. Napier K. L. O'Brien C. P. Childress A. R. (2001). Three decision-making tasks in cocaine-dependent patients: do they measure the same construct? Addiction 96, 1825–1837 10.1046/j.1360-0443.2001.9612182512.x 11784475

[B72] Noël X. Bechara A. Dan B. Hanak C. Verbanck P. (2007). Response inhibition deficit is involved in poor decision making under risk in nonamnesic individuals with alcoholism. Neuropsychology 21, 778–786 10.1037/0894-4105.21.6.778 17983291

[B73] Noël X. Brevers D. Bechara A. (2013). A neurocognitive approach to understanding the neurobiology of addiction. Curr. Opin. Neurobiol. 23, 632–638 10.1016/j.conb.2013.01.018 23395462 PMC3670974

[B74] Oberg S. A. Christie G. J. Tata M. S. (2011). Problem gamblers exhibit reward hypersensitivity in medial frontal cortex during gambling. Neuropsychologia 49, 3768–3775 10.1016/j.neuropsychologia.2011.09.037 21982697

[B75] Palomäki J. Laakasuo M. Salmela M. (2013). ‘This is just so unfair!’: a qualitative analysis of loss-induced emotions and tilting in on-line poker. Int. Gambl. Stud. 13, 255–270 10.1080/14459795.2013.780631

[B76] Persaud N. McLeod P. Cowey A. (2007). Post-decision wagering objectively measures awareness. Nat. Neurosci. 10, 257–261 10.1038/nn1840 17237774

[B77] Peterson E. Moller A. Doudet D. J. Bailey C. J. Vang Hansen K. Rodell A. (2010). Gambling disorder: relation of skin conductance response to dopamine neurotransmission and sensation seeking. Eur. Neurophsychopharmacol. 20, 766–775 10.1016/j.euroneuro.2010.07.010 20813510

[B78] Petry N. M. (2001). Pathological gamblers, with and without substance use disorders, discount delayed rewards at high rates. J Abnorm. Psychol. 110, 482–487 10.1037/0021-843X.110.3.482 11502091

[B79] Petry N. M. Bickel W. K. Arnett M. (1998). Shortened time horizons and insensitivity to future consequences in heroin addicts. Addiction 93, 729–738 10.1046/j.1360-0443.1998.9357298.x 9692271

[B80] Potenza M. N. (2001). The neurobiology of gambling disorder. Semin. Clin. Neuropsychiatr. 6, 217–226 10.1053/scnp.2001.22929 11447573

[B81] Potenza M. N. Steinberg M. A. Skudlarski P. Fulbright R. K. Lacadie C. M. Wilber M. K. (2003). Gambling urges in gambling disorder: a functional magnetic resonance imaging study. Arch. Gen. Psychiatry 60, 828–836 10.1001/archpsyc.60.8.828 12912766

[B82] Power Y. Goodyear B. Crockford D. (2012). Neural correlates of pathological gamblers preference for immediate rewards during the iowa gambling task: an FMRI study. J. Gambl. Stud. 28, 623–636 10.1007/s10899-011-9278-5 22037936

[B83] Rahman S. Sahakian B. J. Cardinal R. N. Rogers R. D. Robbins T. W. (2001). Decision-making and neuropsychiatry. Trends Cogn. Sci. 5, 271–277 10.1016/S1364-6613(00)01650-8 11390298

[B84] Redish A. Jensen S. Johnson A. (2008). Addiction as vulnerabilities in the decision process. Behav. Brain Sci. 31, 461–470 10.1017/S0140525X08004986 18662461 PMC3774323

[B85] Reuter J. Raedler T. Rose M. Hand I. Gläscher J. Büchel C. (2005). Gambling disorder is linked to reduced activation of the mesolimbic reward system. Nat. Neurosci. 8, 147–148 10.1038/nn1378 15643429

[B86] Robbins T. W. Cador M. Taylor J. R. Everitt B. J. (1989). Limbic-striatal interactions in reward-related processes. Neurosci. Biobehav. Rev. 13, 155–162 10.1016/S0149-7634(89)80025-9 2682402

[B87] Robinson T. E. Berridge K. C. (1993). The neural basis of drug craving: an incentive-sensitization theory of addiction. Brain Res. Rev. 18, 247–291 10.1016/0165-0173(93)90013-P 8401595

[B88] Roca M. Torralva T. Lopez P. Cetkovich M. Clark L. Manes F. (2008). Executive functions in pathologic gamblers selected in an ecologic setting. Cogn. Behav. Neurol. 21, 1–4 10.1097/WNN.0b013e3181684358 18327015

[B89] Rogers R. D. Owen A. M. Middleton H. C. Williams E. J. Pickard J. D. Sahakian B. J. (1999). Choosing from small, likely rewards and large, unlikely rewards activated inferior and orbital prefrontal cortex. J. Neurosci. 20, 9029–9038 10516320 10.1523/JNEUROSCI.19-20-09029.1999 PMC6782753

[B90] Rolls E. T. Grabenhorst F. (2008). The orbitofrontal cortex and beyond: from affect to decision-making. Prog. Neurobiol. 86, 216–244 10.1016/j.pneurobio.2008.09.001 18824074

[B91] Rounis E. Maniscalco B. Rothwell J. C. Passingham R. Lau H. (2010). Theta-burst transcranial magnetic stimulation to the prefrontal cortex impairs metacognitive visual awareness. Cogn. Neurosci. 1, 165–175 10.1080/17588921003632529 24168333

[B92] Salimpoor V. N. Benovoy M. Larcher K. Dagher A. Zatorre R. J. (2011). Anatomically distinct dopamine release during anticipation and experience of peak emotion to music. Nat. Neurosci. 14, 257–262 10.1038/nn.2726 21217764

[B93] SAMHSA. (2007). Results from the 2006 National Survey on Drug Use and Health: National Findings (Office of Applied Studies, NSDUH Series H-32, DHHS Publication No. SMA 07-4293), Rockville, MD

[B94] Sato A. Yasuda A. Ohira H. Miyawaki K. Nishikawa M. Kumano H. (2005). Effects of value and reward magnitude on feedback negativity and P300. Neuroreport 16, 407–411 10.1097/00001756-200503150-00020 15729147

[B95] Seguin J. R. Arseneault L. Tremblay R. E. (2007). The contribution of “cool” and “hot” components of decision-making in adolescence: implications for developmental psychopathology. Cogn. Dev. 22, 530–543 10.1016/j.cogdev.2007.08.006

[B96] Sewards T. V. Sewards M. A. (2003). Representations of motivational drives in mesial cortex, medial thalamus, hypothalamus and midbrain. Brain Res. Bull. 61, 25–49 10.1016/S0361-9230(03)00069-8 12788205

[B97] Slachevsky A. Pillon B. Fourneret P. Pradat-Diehl P. Jeannerod M. Dubois B. (2001). Preserved adjustment but impaired awareness in a sensory-motor conflict following prefrontal lesions. J. Cogn. Neurosci. 13, 332–340 10.1162/08989290151137386 11371311

[B98] Slachevsky A. Pillon B. Fourneret P. Renié L. Levy R. (2003). The prefrontal cortex and conscious monitoring of action: an experimental study. Neuropsychologia 41, 655–665 10.1016/S0028-3932(02)00225-7 12591023

[B99] Smith R. S. DeCoster J. (2000). Dual-process models in social and cognitive psychology: conceptual integration and links to underlying memory systems. Pers. Soc. Psychol. Rev. 4, 108–131 10.1207/S15327957PSPR0402_01

[B100] Sonuga-Barke E. Dalen L. Daley D. Remington B. (2002). Are planning, working memory, and inhibition associated with individual differences in preschool ADHD symptoms? Dev. Neuropsychol. 21, 255–272 10.1207/S15326942DN2103_3 12233938

[B101] Stacy A. W. Wiers R. W. (2010). Implicit cognition and addiction: a tool for explaining paradoxical behavior. Annu. Rev. Clin. Psychol. 6, 551–575 10.1146/annurev.clinpsy.121208.131444 20192786 PMC3423976

[B102] Starcke K. Pawlikowski M. Wolf O. T. Altstötter-Gleich C. Brand M. (2011). Decision-making under risk conditions is susceptible to interference by a secondary executive task. Cogn. Process. 12, 177–182 10.1007/s10339-010-0387-3 21210182

[B103] Stocco A. Fum D. Napoli A. (2009). Dissociable processes underlying decisions in the iowa gambling task: a new integrative framework. Behav. Brain Funct. 5:1 10.1186/1744-9081-5-1 19121215 PMC2645419

[B104] Strack F. Deutsch R. (2004). Reflective and impulsive determinants of social behavior. Pers. Soc. Psychol. Rev. 8, 220–247 10.1207/s15327957pspr0803_1 15454347

[B105] Sutherland M. T. McHugh M. J. Pariyadath V. Stein E. A. (2012). Resting state functional connectivity in addiction: Lessons learned and a road ahead. Neuroimage 62, 2281–2295 10.1016/j.neuroimage.2012.01.117 22326834 PMC3401637

[B106] Tanabe J. Thompson L. Claus E. Dalwani M. Hutchison K. Banich M. T. (2007). Prefrontal cortex activity is reduced in gambling and nongambling substance users during decision-making. Hum. Brain Mapp. 28, 1276–1286 10.1002/hbm.20344 17274020 PMC6871281

[B107] Turatto M. Sandrini M. Miniussi C. (2004). The role of the right dorsolateral prefrontal cortex in visual change awareness. Neuroreport 15, 2549–2452 10.1097/00001756-200411150-00024 15538193

[B108] Turnbull O. H. Evans C. E. Bunce A. Carzolio B. O'Connor J. (2005). Emotion-based learning and central executive resources: an investigation of intuition and the Iowa gambling task. Brain Cogn. 57, 244–247 10.1016/j.bandc.2004.08.053 15780457

[B109] van Holst R. J. van den Brink W. Veltman D. J. Goudriaan A. E. (2010). Why gamblers fail to win: a review of cognitive and neuroimaging findings in gambling disorder. Neurosci. Biobehav. Rev. 34, 87–107 10.1016/j.neubiorev.2009.07.007 19632269

[B110] van Holst R. J. Veltman D. J. Büchel C. Van den Brink W. Goudriaan A. E. (2012). Distorted expectancy coding in problem gambling: is the addictive in the anticipation? Biol. Psychiatry 71, 741–748 10.1016/j.biopsych.2011.12.030 22342105

[B111] Verdejo-Garcia A. Bechara A. (2009). A somatic marker theory of addiction. Neuropharmacology 56, 48–62 10.1016/j.neuropharm.2008.07.035 18722390 PMC2635337

[B112] Wardle H. Sproston K. Orford J. Erens B. Griffiths M. Constantine R. (2007). British Gambling Prevalence Survey. London: National Centre for Social Research

[B113] Welte J. W. Barnes G. M. Wieczorek W. F. Tidwell M. C. Parker J. (2002). Gambling participation in the U.S. - results from a national survey. J. Gambl. Stud. 18, 313–337 10.1023/A:1021019915591 12514913

[B114] Whitlow C. T. Liguori A. Livengood L. B. Hart S. L. Mussat-Whitlow B. J. Lamborn C. M. (2004). Long-term heavy marijuana users make costly decisions on a gambling task. Drug Alcohol. Dep. 76, 107–111 10.1016/j.drugalcdep.2004.04.009 15380295

[B115] Wise R. A. Rompre P. P. (1989). Brain dopamine and reward. Annu. Rev. Psychol. 40, 191–225 10.1146/annurev.ps.40.020189.001203 2648975

[B116] Wong S. W. Massé N. Kimmerly D. S. Menon R. S. Shoemaker J. K. (2007). Ventral medial prefrontal cortex and cardiovagal control in conscious humans. Neuroimage 35, 698–708 10.1016/j.neuroimage.2006.12.027 17291781

[B117] Wong S. W. Xue G. Bechara A. (2011). Integrating fMRI with psychophysiological measurements in the study of decision-making. J. Neurosci. Psychol. Econ. 4, 85–94 10.1037/a0023525 21818407 PMC3147053

[B118] Yeung N. Botvinick M. M. Cohen J. D. (2004). The neural basis of error detection: Conflict monitoring and the error-related negativity. Psychol. Rev. 111, 931–959 10.1037/0033-295X.111.4.931 15482068

[B119] Yi S. Kanetkar V. (2010). Implicit measures of attitudes toward gambling: an exploratory study. J. Gambl. Issues 24, 140–163 10.4309/jgi.2010.24.9

[B120] Zack M. Poulos C. X. (2004). Amphetamine primes motivation to gamble and gambling-related semantic networks in problem gamblers. Neuropsychopharmacology 29, 195–207 10.1038/sj.npp.1300333 14571257

[B121] Zelazo P. D. Müller U. (2002). Executive function in typical and atypical development, in Handbook of Childhood Cognitive Development, ed Goswami U. (Oxford: Blackwell), 445–469

